# *Actinobacillus pleuropneumoniae* FliY and YdjN are involved in cysteine/cystine utilization, oxidative resistance, and biofilm formation but are not determinants of virulence

**DOI:** 10.3389/fmicb.2023.1169774

**Published:** 2023-05-12

**Authors:** Fan Zhao, Huan Xu, Yubing Chen, Juan Xiao, Miao Zhang, Zhuo Li, Jinlin Liu, Chao Qi

**Affiliations:** Hubei Key Laboratory of Genetic Regulation and Integrative Biology, College of Life Sciences, Central China Normal University, Wuhan, Hubei, China

**Keywords:** *Actinobacillus pleuropneumoniae*, FliY/YdjN, cysteine/cystine acquisition, oxidative tolerance, biofilm formation, virulence

## Abstract

**Introduction:**

*Actinobacillus pleuropneumoniae* (*A. pleuropneumoniae*) is a member of *Actinobacillus* in family Pasteurellaceae. It is the causative agent of porcine pleuropneumonia, which has caused huge economic losses to pig industry over the world. Cysteine is a precursor of many important biomolecules and defense compounds in the cell. However, molecular mechanisms of cysteine transport in *A. pleuropneumoniae* are unclear.

**Methods:**

In this study, gene-deleted mutants were generated and investigated, to reveal the roles of potential cysteine/cystine transport proteins FliY and YdjN of *A. pleuropneumoniae*.

**Results:**

Our results indicated that the growth of *A. pleuropneumoniae* was not affected after *fliY* or *ydjN* single gene deletion, but absence of both FliY and YdjN decreased the growth ability significantly, when cultured in the chemically defined medium (CDM) supplemented with cysteine or cystine as the only sulfur source. *A. pleuropneumoniae* double deletion mutant Δ*fliY*Δ*ydjN* showed increased sensitivity to oxidative stress. Besides, *trans*-complementation of YdjN into Δ*fliY*Δ*ydjN* and wild type leads to increased biofilm formation in CDM. However, the virulence of Δ*fliY*Δ*ydjN* was not attenuated in mice or pigs.

**Discussion:**

These findings suggest that *A. pleuropneumoniae* FliY and YdjN are involved in the cysteine/cystine acquisition, oxidative tolerance, and biofilm formation, but not contribute to the pathogenicity of *A. pleuropneumoniae*.

## Introduction

Porcine pleuropneumonia caused by *A. pleuropneumoniae* is associated with extensive morbidity and mortality in growing pigs; therefore, it is of particular importance for the prevention and control of this disease (Sassu et al., 2018). The current knowledge has indicated that several virulence factors are involved in adherence and colonization, immune escape, and tissue damage during the pathogenesis of *A. pleuropneumoniae* infection, including but not limited to Apx toxins, lipopolysaccharides, capsular polysaccharides, adhesins, iron acquisition factors, enzymes, biofilm formation, and signal transduction systems (Chiers et al., 2010). However, more efforts are still needed for a comprehensive understanding of the pathogenesis of *A. pleuropneumoniae* infection.

After invading the host, the acquisition of essential nutrients is critical for the survival and successful infection of pathogenic bacteria. The mechanisms involved in nutritional satisfaction are essential for bacterial pathogenicity (Núñez et al., 2018). Sulfur is a key constituent of sulfur-containing amino acids, coenzymes, vitamins, nucleotides, and peptides (Beinert, 2000). Most of the sulfur metabolic pathways are unique to microorganisms and are a potential target for future therapeutic intervention against pathogen attacks (Paritala and Carroll, 2013). The transporter protein Sbp in *A. pleuropneumoniae* has been found to be associated with sulfate and methionine utilization, but it was not found to be a determinant of virulence in a previous study by Gao et al. (2020). More efforts are needed for the mechanisms of sulfur assimilation and metabolism in *A. pleuropneumoniae*.

As one of the important sulfur sources, cysteine is used for the biosynthesis of a variety of cellular components, such as protein, glutathione, biotin, and more (Hicks and Mullholland, 2018). In addition, cysteine has been implicated in the survival and virulence of pathogens (Soutourina et al., 2009; Méndez et al., 2011). In *Escherichia coli*, the ABC transporter FliY was reported to be essential for the import of cysteine and trace cystine, whereas YdjN is a predominant importer of cystine when cystine is more abundant (Imlay et al., 2015). Two homologous proteins, namely, APJL_1690 (FliY) and APJL_0600 (YdjN), have been identified from *A. pleuropneumoniae* previously (Gao et al., 2020). Therefore, the roles of these two potential cysteine/cystine transporters in the sulfur acquisition, stress tolerance, biofilm formation, and virulence of *A. pleuropneumoniae* were investigated in this study.

## Materials and methods

### Bacterial strains, primers, plasmids, and growth conditions

The bacterial strains, plasmids, and primers used in this study are listed in Table 1. *Actinobacillus pleuropneumoniae* strains were cultured in tryptic soy agar (TSA, Dickinson and Company, Franklin Lakes, NJ, United States) or tryptic soy broth (TSB), supplemented with 10 μg/ml nicotinamide adenine dinucleotide (NAD^+^; Sigma, St. Louis, MO, United States) and 10% fetal calf serum (Gibco BRL, Grand Island, NY, United States), and 2 μg/ml chloramphenicol and 5% (m/v) sucrose were added for the selection of *A. pleuropneumoniae* single-crossover mutants and double-crossover mutants, respectively. To select the complementation strain, 2 μg/ml chloramphenicol was added. To evaluate the utilization of different sulfur sources, *A. pleuropneumoniae* strains were grown in chemically defined media (CDM), which consisted of different sulfur sources (potassium sulfate, cysteine, cystine, glutathione, and methionine). The preparation of CDM was carried out as described previously (Herriott et al., 1970). *Escherichia coli* strains were grown on Luria–Bertani (LB) agar (Oxoid, Basingstoke, Hants, UK) or in LB broth, supplemented with ampicillin (100 μg/ml) and/or diaminopimelic acid (DAP; 50 μg/ml) if needed. Unless otherwise noted, bacteria in liquid media were cultured in a common incubator at 37°C with shaking (200 rpm), and bacteria on agar plates were incubated statically at 37°C.

**Table 1:** Bacterial strains, plasmids, and primers used in this study.

**Strains, plasmids, and primers**	**Relevant characteristics**	**Sources**
***Actinobacillus pleuropneumoniae***		
WF83	Serovar 7, wild type (WT)	From Dr. Pat Blackall
Δ*fliY*	*A. pleuropneumoniae* WF83 *fliY*-deletion mutant	This work
Δ*ydjN*	*A. pleuropneumoniae* WF83 *ydjN*-deletion mutant	This work
Δ*fliY*Δ*ydjN*	*A. pleuropneumoniae* WF83 *fliY* and *ydjN* double-deletion mutant.	This work
Δ*fliY*Δ*ydjN*-*fliY^+^*	*A. pleuropneumoniae* Δ*fliY*Δ*ydjN* complemented with *fliY* gene	This work
Δ*fliY*Δ*ydjN*-*ydjN^+^*	*A. pleuropneumoniae* Δ*fliY*Δ*ydjN* complemented with *ydjN* gene	This work
WT-*ydjN^+^*	*A. pleuropneumoniae* WT containing complementation plasmid pJFF-*ydjN*	This work
***Escherichia coli***		
DH5a	Cloning vehicle: *supE44* Δ*lacU169* (*ϕ80 lacZ*ΔM15) *hsdR17 recA1 endA1 gyrA96 thi-1 relA1*	Takara, Dalian, China
β2155	Transconjugation donor: *thrB1004 pro thi strA hsdS lacZ* Δ*M15 (F'lacZ* Δ*M15lacI^*q*^ traD36 proA^+^ proB^+^) dap* : : *erm recA* : : *RP4-2-tet* : : *Mu-km λpir, Erm^*r*^ Tet^*r*^ Kan ^*r*^*	Oswald et al., 1999
**Plasmids**		
pEMOC2	Transconjugation vector: ColE1 *ori mob* RP4 *sacB, amp^*r*^ cm^*r*^*	Baltes et al., 2002
pEMOC2-Δ*fliY*	Up and downstream arms of *fliY* gene were ligated into pEMOC2, and used as the transconjugation vector for *fliY* gene deletion	This work
pEMOC2-Δ*ydjN*	Up and downstream arms of *ydjN* gene were ligated into pEMOC2, and used as the transconjugation vector for *ydjN* gene deletion	This work
pJFF224-XN	*E. coli*-APP shuttle vector: RSF1010 replicon; *mob oriV, cm^*r*^*	Frey, 1992
pJFF-*fliY*	pJFF224-XN carrying the intact *fliY* gene of *A. pleuropneumoniae* WF83 and used for the construction of complementation strain	This work
pJFF-*ydjN*	pJFF224-XN carrying the intact *fliY* gene of *A. pleuropneumoniae* WF83 and used for the construction of complementation strain	This work
**Primers**		
*fliY*-F1	5′-TTTGTCGACTTATATTACCCGACTGACGGCTAC-3′, forward primer with *Sal*I site (underlined) comprising position −976 to −953 of the *fliY* coding sequence	This work
*fliY*-R1	5′-CGCTGATAACGAGGCATTTTATTTATTTCAAGTAGGAATCCTCTTAATTGGAGAAA-3′, reverse primer comprising position +813 to +784 and −1 to −26 of the *fliY* coding sequence	
*fliY*-F2	5′-TTTCTCCAATTAAGAGGATTCCTACTTGAAATAAATAAAATGCCTCGTTATCAGCG-3′, forward primer comprising position −26 to −1 and +784 to +813 of the *fliY* coding sequence	This work
*fliY*-R2	5′-TTTGCGGCCGCTCTCAAGTTGTCAGGCGATAATCT-3′, reverse primer with *Not*I site (underlined) comprising position +1,749 to +1,726 of the *fliY* coding sequence	
*fliY*-F3	5′-TTGTCGACATTGCTGTGGCGCCTATTTAGCCT-3′, upstream primer with *Sal*I site (underlined) comprising position −238 to −215 of *fliY*. This primer was used to clone *fliY* gene for construction of complementation strain	This work
*fliY*-R3	5′-TTGCGGCCGCTTATTTCACGCTAATATCACGACC-3′, downstream primer with *Not*I site (underlined) comprising position +783 to +760 of *fliY*	
*fliY*-F4	5′-CGGCGTTAGTGGTACGAAAT-3′, upstream primer comprising position +371 to +390 of *fliY*. This primer was used to validate the presence of *fliY* gene in the mutant	This work
*fliY*-R4	5′-CCAATTGCCGCACTAATTTT-3′, downstream primer comprising position +710 to +691 of *fliY*	
*ydjN*-F1	5′-TTTGTCGACAAGCGCATGATATTACCGATTCGT-3′, forward primer with *Sal*I site (underlined) comprising position −998 to −975 of the *ydjN* coding sequence	This work
*ydjN*-R1	5′-AAATTTGCCAAAAATCACCGCTTGCCGAACCTCCACACAATAAAAAAG-3′, reverse primer comprising position +1,350 to +1,327 and −1 to −24 of the *ydjN* coding sequence	
*ydjN*-F2	5′-CTTTTTTATTGTGTGGAGGTTCGGCAAGCGGTGATTTTTGGCAAATTT-3′, forward primer comprising position −24 to −1 and +1,327 to +1,350 of the *ydjN* coding sequence	This work
*ydjN*-R2	5′-TTTGCGGCCGCTAGGTGTTGCATTACCTATCCTCT-3′, reverse primer with *Not*I site (underlined) comprising position +2,384 to +2,361 of the *ydjN* coding sequence	
*ydjN*-F3	5′-TTGTCGACCGGAGAATGGAAATTAGCACA-3′, upstream primer with *Sal*I site (underlined) comprising position −195 to −175 of *ydjN*. This primer was used to clone *ydjN* gene for construction of complementation strain	This work
*ydjN*-R3	5′-TTGCGGCCGCTTATGCTTGATTAAGCCACTTGT-3′, downstream primer with *Not*I site (underlined) comprising position +1,326 to +1,304 of *ydjN*	
*ydjN*-F4	5′-CGAAACGCAAACCGATAAAT-3′, upstream primer comprising position +933 to +952 of *ydjN*. This primer was used to validate the presence of *ydjN* gene in the mutant	This work
*ydjN*-R4	5′-GCACTACAATCGCTGCAAAA-3′, downstream primer comprising position +1,183 to +1,164 of *ydjN*	
*ydjN*-F5	5′-ATAGCGAACTTTCCCAAACG-3′, upstream primer comprising position −331 to −312 of *ydjN*. This primer was used to validate the absence of *ydjN* gene in the mutant	This work
*ydjN*-R5	5′-AAGCGAACGCACCTTATTTG-3′, downstream primer comprising position +1,695 to +1,676 of *ydjN*	
ApxIV-F	5′-CAGAATCAAACTTTCGGCG-3′, forward primer specific for *apxIVA* gene of *A. pleuropneumoniae*, and used to confirm bacterial colonies isolated from animals are *A. pleuropneumoniae*	Schaller et al., 2001
ApxIV-R	5′-GCACAAGGTAAAACGGTGA-3′, reverse primer specific for *apxIVA* gene of *A. pleuropneumoniae*	

### DNA manipulations

The target genes *fliY* and *ydjN* were knocked out sequentially from *A. pleuropneumoniae* wild-type (WT) strain WF83 (serovar 7) using one-step transconjugation methods, as described previously (Oswald et al., 1999). For the construction of a single-gene deleted mutant, two homologous arms of the target gene were amplified by PCR separately and ligated together using overlap PCR, then ligated into the transconjugation plasmid pEMOC2 (Baltes et al., 2002), to generate the recombination vector, and then transformed into *E. coli* β2155 to form the donor cells. The recombination vector was introduced into *A. pleuropneumoniae* WT by co-cultivation of the WT and donor cells. After chloramphenicol-mediated positive selection and sucrose-mediated counter-selection, chloramphenicol-sensitive and sucrose-resistant colonies were selected and recognized as target mutants and then verified with PCR and sequencing at Sangon Biotech (Shanghai, China). The double-gene deletion mutant was generated by using the single-gene deletion mutant as a parent.

The complemented mutants were constructed as described previously (Liu et al., 2018). The intact *fliY* and *ydjN* genes were cloned from *A. pleuropneumoniae* WT and inserted into the *E. coli*–*A. pleuropneumoniae* shuttle vector, pJFF224-XN (Frey, 1992), to generate the plasmids pJFF-*fliY* and pJFF-*ydjN* separately and transformed into Δ*fliY*Δ*ydjN* by electroporation. Chloramphenicol-resistance colonies were selected and verified by PCR and RT-PCR.

### Growth ability and sulfur source utilization assays

To assess the role of *fliY* and *ydjN* on bacterial growth, *A. pleuroPneumoniae* WT, the single-gene deletion mutants Δ*fliY* and Δ*ydjN*, and the double-gene deletion mutant Δ*fliY*Δ*ydjN* were grown separately in TSB. Overnight cultures were separately diluted (1:100) in 5 ml fresh TSB in 20 ml glass tubes and cultured with shaking for 10 h. The optical densities at 600 nm (OD_600_) were measured each hour, so as to generate bacterial growth curves. OD_600_ values were repeated in triplicate with at least three independent experiments.

To analyze the roles of the target genes in the utilization of sulfur sources, overnight cultures of each strain were inoculated in CDM with different sulfur sources, including complete CDM (containing potassium sulfate, methionine, glutathione, cysteine, and cystine), blank CDM (without sulfur), and CDM with only one type of sulfur source, and the growth curves were obtained.

### Oxidative stress sensitivity assay

In order to analyze the effect of FliY and YdjN on the defense of *A. pleuropneumoniae* against H_2_O_2_-induced oxidative stress, overnight cultures were diluted (1:100) in fresh TSB separately and grown to the mid-log phase. Samples were taken, centrifuged, washed with PBS (phosphate-buffered saline, pH7.4), and re-suspended in PBS or PBS with H_2_O_2_ (2.5 mM), then incubated at 37°C. Samples were taken at 1 h and 2 h after treatment, diluted immediately in PBS, and spread onto TSA plates. After incubation, viable counts were determined by plate counts on the agar. The survival rate of each strain was calculated by dividing viable counts exposed to H_2_O_2_ by the negative control (without H_2_O_2_ treatment). An oxidative stress sensitivity assay was performed at least in triplicate.

### Biofilm formation assay

The biofilm formation of *A. pleuropneumoniae* strains was measured as described previously but with slight modifications (Liu et al., 2017). In brief, *A. pleuropneumoniae* overnight cultures were diluted (1:100) in 1 ml of TSB or complete CDM in 2 ml tubes and added into 24-well flat-bottom polystyrene plates separately. The plates were incubated at 37°C for 24 h statically. Each well was washed twice with 1 ml of sterile water and stained with 500 μl of 0.4% crystal violet for 15 min, washed with water three times, and then air-dried for 30 min. Acetic acid (33%, 1 ml) was added to each well and shaken for 15 min to quantify the number of biofilms. The absorbance was measured at 590 nm (OD_590_) and normalized to the OD_600_ values of cultures to account for growth differences. To better reveal the influence of these two potential cysteine/cystine transporters, FliY and YdjN, on the biofilm formation of *A. pleuropneumoniae*, the florescent stain was used for the visualization of biofilm. In brief, sterile cover glasses were pre-put at the bottom of 24-well plates, and bacterial cultures were diluted (1:100) in 1 ml of complete CDM and added to 24-well plates. The plates were incubated at 37°C for 24 h. After two washes with 1 ml of PBS, 1 ml of 1.5 μM SYTO 9 was added to each well and stained for 20 min in the dark. After washing with PBS, the cover glasses were taken out and placed on slide glasses, and fluorescence signals were analyzed with a confocal laser scanning microscope (CLSM, Axio Scope, Carl Zeiss Meditec, Berlin, Germany). For CLSM analysis, the excitation light wavelength was set at 488 nm and collected with a 495–525 nm bandpass filter. ImageJ software was used for image processing.

### Bacterial colonization assay

Bacterial colonization in the lung tissues of mice was carried out as described previously (Liu et al., 2018). Fifteen 6-week-old female BALB/c mice (purchased from Hubei Provincial Center for Disease Control and Prevention, Wuhan, China) were divided into three groups of five mice per group. Groups I and II were inoculated intraperitoneally with 2.5 × 10^7^ CFU of *A. pleuropneumoniae* WT and Δ*fliY*Δ*ydjN* in 0.5 ml saline, respectively, and group III was inoculated with 0.5 ml saline and used as a negative control. The mice were monitored closely, and severely diseased mice (designated as those with dyspnea and depression) were euthanized. The surviving mice were euthanized 24 h after inoculation. Lung tissues (~0.1 g) were collected aseptically for homogenization. The lung homogenates were diluted properly, spread on TSA plates, and incubated at 37°C overnight. Single colonies were verified by PCR with primers *apxIVA*-F/*apxIVA*-R (specific for *apxIVA* gene) (Schaller et al., 2001), and the bacterial load of each strain was calculated. The animal experiments in this study were approved by the Animal Ethics Committee at the Central China Normal University and carried out in accordance with the guidelines for the Care and Use of Laboratory Animals provided by this committee (Ethics Ratification ID: CCNU-IACUC-2020-015).

### Experimental infection in pigs

The pig infection assay (*in vivo* competitive colonization) was performed as described previously, with minor modifications (Beddek et al., 2004), so as to determine the virulence of *A. pleuropneumoniae* Δ*fliY*Δ*ydjN*. Bacteria cultures at the mid-log phase were sampled and diluted to ~2.0 × 10^8^ CFU/ml, respectively, and then mixed in an equal volume. Five newborn piglets were purchased from an *A. pleuropneumoniae*-free herd (negative in serological and etiological tests) and inoculated intranasally with 1 ml of the mixed bacterial culture. Animals were monitored every 6 h for 24 h, and severely diseased pigs, designated as those with dyspnea, depression, or with a rectal temperature lower than 38.5°C, were humanely euthanized. The surviving pigs were humanely euthanized at 24 h after infection. Lung tissues were obtained aseptically for homogenization. The lung tissue homogenates were diluted properly and spread onto TSA plates, then cultured at 37°C overnight. For each infected piglet, at least 100 single colonies were picked randomly and cultured in TSB separately. PCR assays with primers specific to the *apxIVA* gene (*apxIVA*-F/*apxIVA*-R), *fliY* gene (*fliY*-F4/*fliY*-R4), and *ydjN* gene (*ydjN*-F4/*ydjN*-R4) were used to differentiate these re-isolates. *Actinobacillus pleuropneumoniae* WT was positive in all three PCR assays, whereas Δ*fliY*Δ*ydjN* was positive in *apxIVA* but negative in *fliY* or *ydjN* gene amplification. The *in vivo* competitive index (CI) values were calculated by dividing the ratio of Δ*fliY*Δ*ydjN* to WT (output) by the ratio of Δ*fliY*Δ*ydjN* to WT in the bacteria mixture (input).

### Statistical analysis

Unless otherwise specified, data obtained from the present study were expressed as the mean ± SD and analyzed by a two-tailed unpaired Student's *t*-test. A *P*-value of < 0.05 was considered significant, and a *P*-value of < 0.01 was considered highly significant.

## Results

### Generation of *A. pleuropneumoniae* gene deletion mutants and complementation

The target genes *fliY* and *ydjN* were knocked out from the *A. pleuropneumoniae* WT using one-step transconjugation methods, resulting in the single-gene deletion mutants Δ*fliY* and Δ*ydjN* and double-gene deletion mutant Δ*fliY*Δ*ydjN*, and the absence of *fliY* and *ydjN* in these mutants was verified with PCRs and DNA sequencing (Supplementary Figure S1). We next constructed corresponding complementation strains based on the *E. coli*–*A. pleuropneumoniae* shuttle vector pJFF224-XN. The complementary plasmids pJFF-*fliY* and pJFF-*ydjN* were transformed into Δ*fliY*Δ*ydiN*, generating Δ*fliY*Δ*ydiN*-*fliY*^+^ and Δ*fliY*Δ*ydiN*-*ydiN*^+^, respectively. These mutants were screened by chloramphenicol resistance, and then verified by PCR and reverse transcription PCR (RT-PCR) assays with primers specific for target genes (Supplementary Figure S2). In addition, pJFF-*ydjN* was transferred into the WT to generate WT-*ydiN*^+^.

### The FliY and YdjN double mutation leads to a longer lag phase in TSB

**Figure 1:**
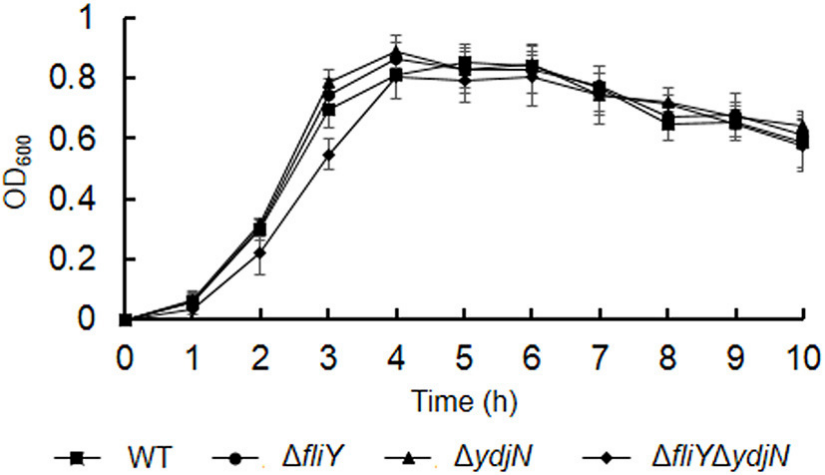
Growth curves of *Actinobacillus pleuropneumoniae* WT and mutants in TSB. Data are expressed as the mean ± SD of at least three independent experiments.

The growth curves of *A. pleuropneumoniae* WT and mutants in TSB were obtained. As shown in Figure 1, the *fliY* and *ydjN* single-gene deletion mutants (Δ*fliY* and Δ*ydjN*) showed similar growth levels to that of the WT. Though the double-gene deletion mutant Δ*fliY*Δ*ydjN* exhibited similar growth levels relative to that of the WT at stationary phase, the growth levels of Δ*fliY*Δ*ydjN* at 2 h (*P*-value < 0.05) and 3 h (*P*-value < 0.01) were significantly lower than those of the WT, indicating a longer lag phase of the FliY and YdjN double mutant.

### FliY and YdjN participate in the acquisition of sulfur sources

**Figure 2:**
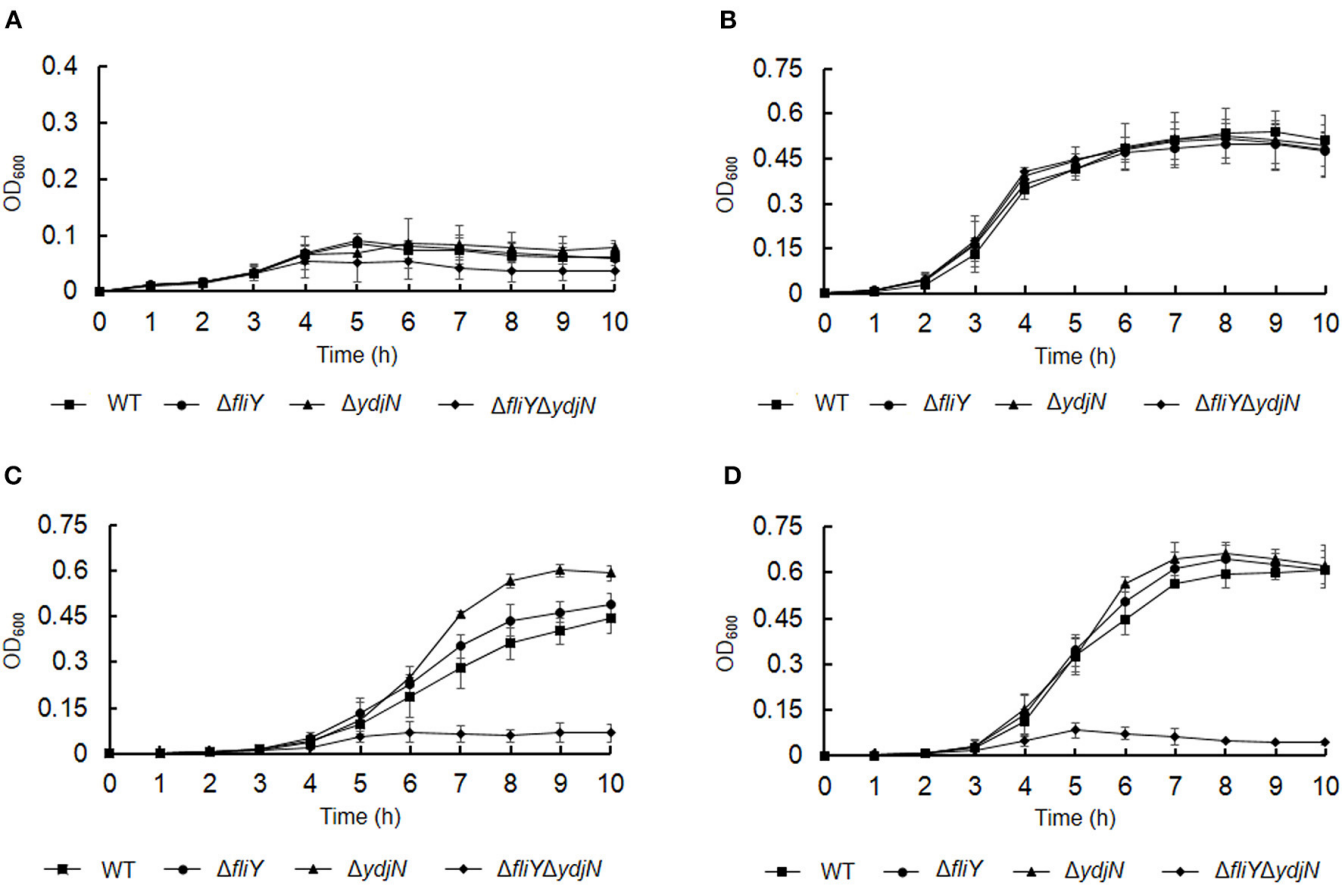
Proliferation of *Actinobacillus pleuropneumoniae* WT and mutants in CDM supplemented with different sulfur sources. *Actinobacillus pleuropneumoniae* WT, single-gene deletion mutants, Δ*fliY* and Δ*ydiN*, and double-gene deletion mutant, Δ*fliY*Δ*ydiN*, were grown in the blank CDM **(A)**, complete CDM **(B)**, and CDM with cysteine **(C)** or cystine **(D)** as the sole sulfur source, separately. Data are expressed as the mean ± SD of at least three independent experiments.

**Figure 3:**
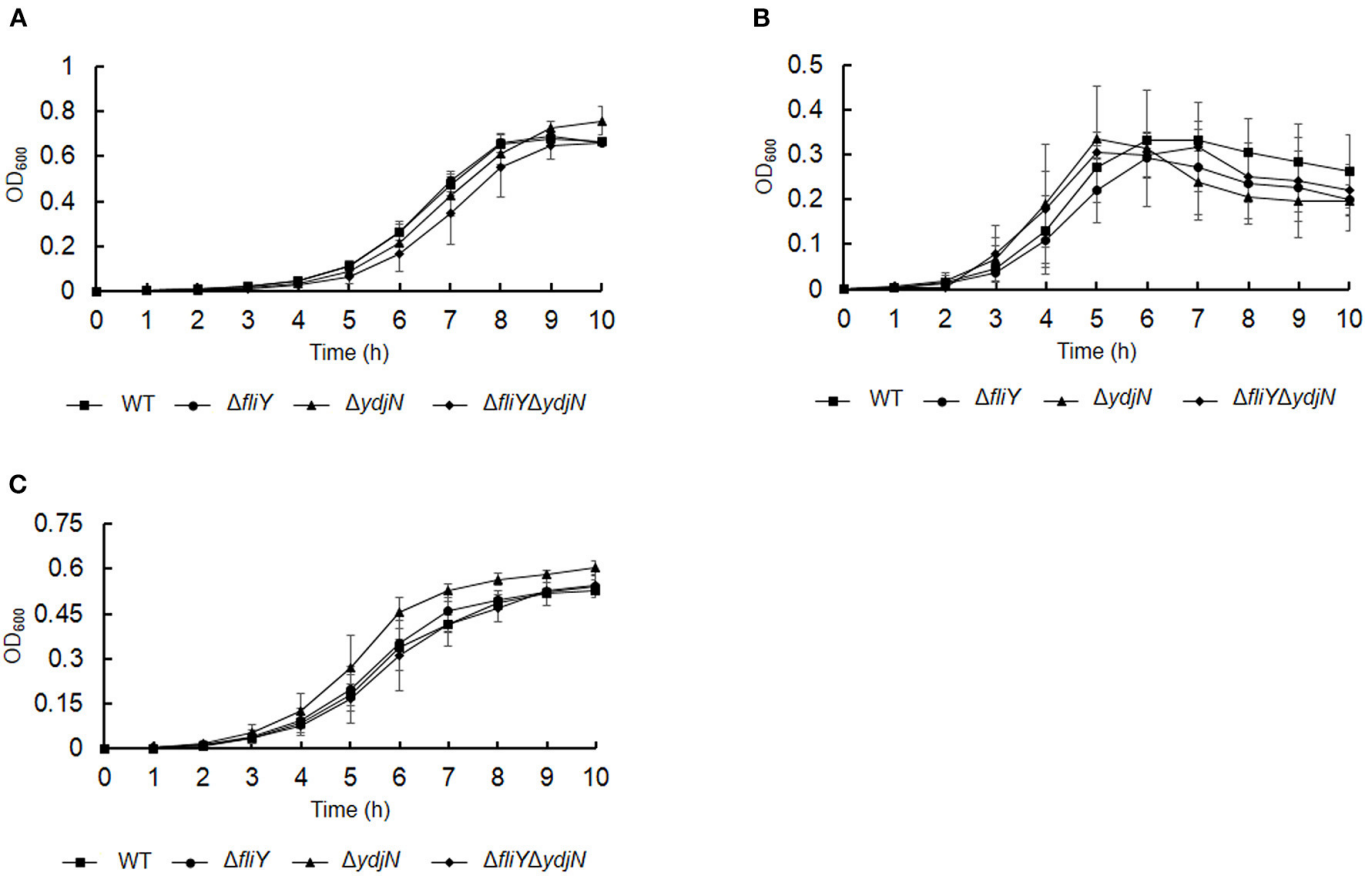
Growth of *Actinobacillus pleuropneumoniae* WT and mutants in CDM supplemented with potassium sulfate **(A)**, methionine **(B)**, and glutathione **(C)** as the sole source of sulfur. Error bars represent ± 1 standard error of the mean. The average of at least three independent trials is presented.

**Figure 4:**
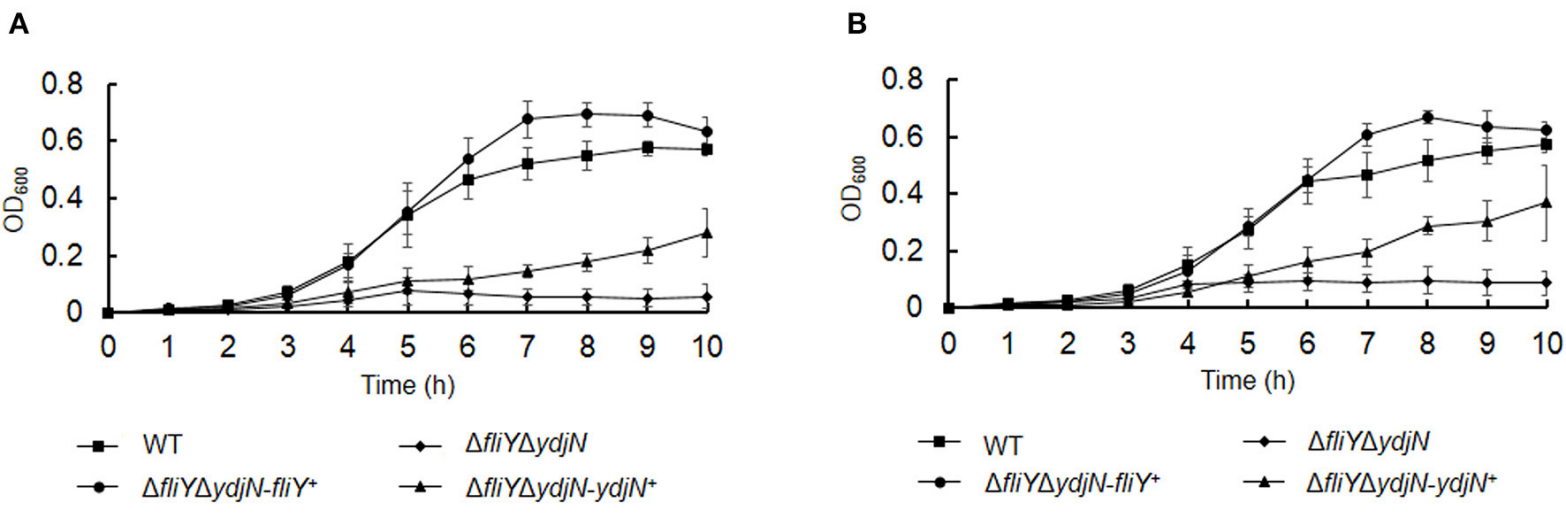
Restoration of growth phenotypes of *Actinobacillus pleuropneumoniae* via *trans*-complementation. *Actinobacillus pleuropneumoniae* WT, double-gene deletion mutant, Δ*fliY*Δ*ydiN*, and complementation strains, Δ*fliY*Δ*ydjN*-*fliY*^+^ and Δ*fliY*Δ*ydjN*-*ydjN*^+^, were grown in the CDM with cysteine **(A)** or cystine **(B)** as the sole sulfur source. Data are expressed as the mean ± SD of at least three independent experiments.

The roles of FliY and YdjN in the acquisition of sulfur source(s) were investigated by the cultivation of *A. pleuropneumoniae* WT and mutants in sulfur-limited CDM. As shown in Figure 2, all these bacterial strains showed no or little growth in sulfur-free CDM (Figure 2A), and there was no significant difference in the growth of *A. pleuropneumoniae* WT and mutants in complete CDM (Figure 2B), as well as in CDM supplemented with potassium sulfate, methionine, or glutathione as the sole sulfur source (Figure 3). When cysteine or cystine was used as the only sulfur source, the growth capacity of Δ*fliY*Δ*ydjN* was significantly impaired (Figures 2C, D). Surprisingly, the single-gene deletion mutants Δ*fliY* and Δ*ydjN* did not show any decreased growth levels compared to the WT strain in CDM supplemented with cysteine or cystine (Figures 2C, D). The complementation of Δ*fliY*Δ*ydjN* with *fliY* restored the growth defect to the WT level, while the transformation of *ydjN* rescued the growth defect of Δ*fliY*Δ*ydjN*, though not yet back to the WT level (Figure 4). These results suggest that FliY and YdjN are required for the utilization of cysteine and cystine as sulfur sources in *A. pleuropneumoniae*.

### Deletion of FliY and YdjN increases susceptibility to oxidative stress

**Figure 5:**
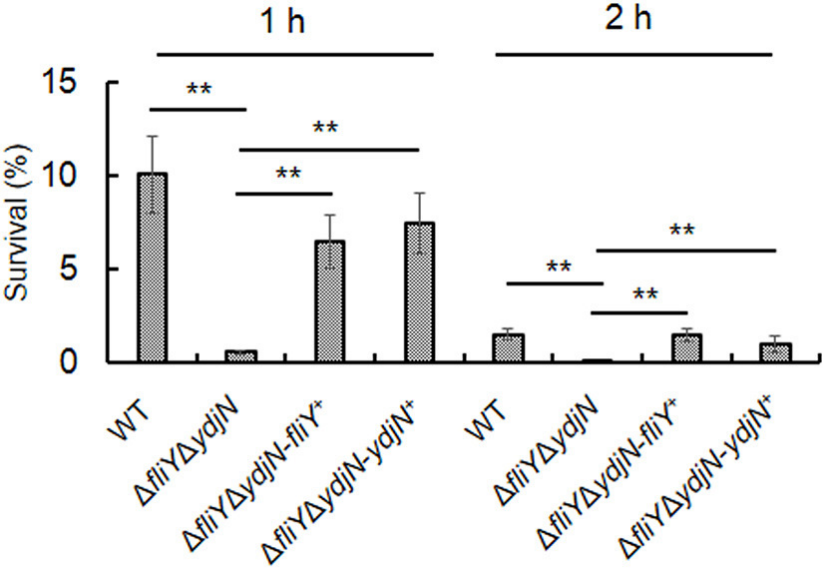
Survival of *Actinobacillus pleuropneumoniae* following exposure to oxidative stress. *Actinobacillus pleuropneumoniae* WT, Δ*fliY*Δ*ydjN*, and complemented strains, Δ*fliY*Δ*ydjN*-*fliY*^+^ and Δ*fliY*Δ*ydjN*-*ydjN*^+^, at mid-log phase were washed and re-suspended in PBS with 2.5 mM H_2_O_2_, incubated at 37°C, and then sampled at 1 h and 2 h. Bacterial viability was measured by plate counting. The survival rate was calculated by dividing the CFUs of H_2_O_2_-treated bacterial suspension by the CFUs of the untreated control. The double asterisk (**) indicates a highly significant difference (*P*-value < 0.01).

The sensitivity of *A. pleuropneumoniae* WT and Δ*fliY*Δ*ydjN* to H_2_O_2_-induced oxidative stress was determined. As shown in Figure 5, Δ*fliY*Δ*ydjN* was more sensitive to 2.5 mM H_2_O_2_ when compared to the WT strain (*P*-value < 0.01), the average survival rates of Δ*fliY*Δ*ydjN* and WT were 0.58% (0.58 ± 0.11%) and 10.08% (10.08 ± 2.08%) 1 h after exposure to H_2_O_2_, respectively, and the survival rates of Δ*fliY*Δ*ydjN* and WT were 0.014% (0.014 ± 0.0030%) and 1.53% (1.53 ± 0.28%) at 2 h after exposure to H_2_O_2_, respectively. The decreased oxidative stress tolerance of Δ*fliY*Δ*ydjN* was recovered by complementation with the *fliY* or *ydjN* gene. These data indicate the role of FliY and YdjN in conferring resistance against oxidative stress in *A. pleuropneumoniae*.

### YdjN regulates *A. pleuropneumoniae* biofilm formation

**Figure 6:**
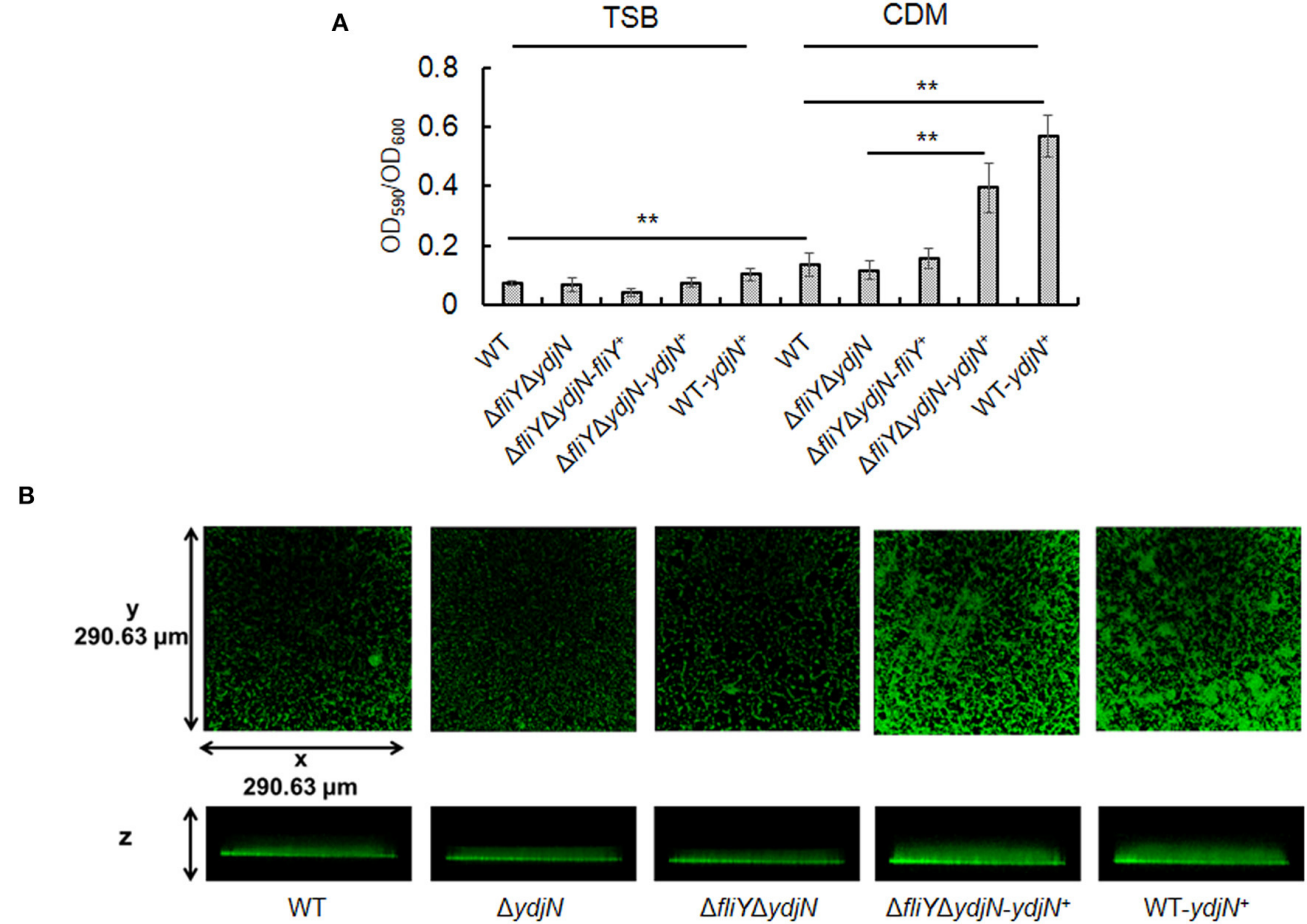
Complementation of the *ydjN* gene increases biofilm formation. **(A)** *Actinobacillus pleuropneumoniae* biofilms were evaluated by CV staining. *Actinobacillus pleuropneumoniae* WT and mutant strains were cultured in TSB or complete CDM. The biofilm biomass (OD_590_) was determined by the CV staining method and normalized to the bacterial growth (OD_600_). Data from at least three biological replicates were averaged, and the statistical significance was determined. **indicates significance with a *P*-value < 0.01. **(B)** *Actinobacillus pleuropneumoniae* biofilms observed by CSLM. *Actinobacillus pleuropneumoniae* WT and mutant strains were grown in complete CDM, as described in Materials and methods. After 24-h growth, the biofilms were washed and stained with SYTO 9 and scanned by CLSM with a Plan-Neofluar × 10/0.3 objective lens. Representative orthogonal views from three independent experiments are displayed.

The possible impacts of FliY and YdjN on the *A. pleuropneumoniae* biofilm formation were investigated. As shown in Figure 6A, *A. pleuropneumoniae* WT formed more biofilm in CDM compared to that in TSB (*P*-value < 0.01). Notably, *A. pleuropneumoniae* strains containing plasmid pJFF-*ydjN* (Δ*fliY*Δ*ydjN*-*ydjN*^+^ and WT-*ydjN*^+^) showed significantly more biofilm than their parental strains Δ*fliY*Δ*ydjN* and WT (*P*-value < 0.01), respectively. The enhanced biofilm formation of Δ*fliY*Δ*ydjN*-*ydjN*^+^ and WT-*ydjN*^+^ was also observed under a confocal scanning laser microscope (Figure 6B). These results indicate that the complementation of the *ydjN* gene enhances the biofilm formation of *A. pleuropneumoniae*.

### FliY and YdjN are not required for the colonization of *A. pleuropneumoniae* in mice

The requirement of FliY/YdjN for cysteine/cystine-dependent growth and oxidative stress resistance *in vitro* prompted us to investigate the effect of these two proteins on the virulence of *A. pleuropneumoniae*. We assessed the colonization ability of the double-gene deletion mutant Δ*fliY*Δ*ydjN* in mice. Mice infected with the Δ*fliY*Δ*ydjN* showed similar bacterial loads (logCFU/g, 6.82 ± 0.94), with those infected with *A. pleuropneumoniae* WT (logCFU/g, 7.37 ± 0.90; *P*-value > 0.05). No bacteria were re-isolated in the healthy control group. This result suggests that the deletion of *fliY* and *ydjN* does not affect the colonization ability of *A. pleuropneumoniae* in the lungs of mice.

### FliY and YdjN do not contribute to the virulence of *A. pleuropneumoniae* in pigs

The virulence of Δ*fliY*Δ*ydjN* was further assessed in a pig infection model. As shown in Table 2, the input ratio of Δ*fliY*Δ*ydjN* to WT was calculated as 1.09, and the average output ratio was calculated to be 0.63 (Table 2). Thus, the average CI value between Δ*fliY*Δ*ydjN* and WF83 was 0.58, which was higher than the cutoff value (0.2) for attenuation (Beddek et al., 2004). This result indicates that Δ*fliY*Δ*ydjN* was still virulent in pigs, which is consistent with the result of the mice infection assay, and FliY and YdjN do not contribute to the virulence of *A. pleuropneumoniae*.

**Table 2:** Competitive indices of *Actinobacillus pleuropneumoniae* Δ*fliY*Δ*ydjN* and WT in pigs.

**Pigs^a^**	**Input (CFU)** ^**b**^			**Lung (CFU)** ^**c**^			**Output/Input**
	Δ***fliY***Δ***ydjN***	**WT**	Δ***fliY***Δ***ydjN*****/WT**	Δ***fliY***Δ***ydjN***	**WT**	Δ***fliY***Δ***ydjN*****/WT**	
1	1.20 × 10^8^	1.10 × 10^8^	1.09	38	61	0.62	0.57
2				46	49	0.94	0.86
3				47	58	0.81	0.74
4				28	77	0.36	0.33
5				29	71	0.41	0.38
CI value^d^				0.58			

^a^The *in vivo* CI assay was performed based on methods described previously (Beddek et al., 2004), with a minor modification.

^b^A. pleuropneumoniae WT and ΔfliYΔydjN at the mid-log phase were diluted in PBS separately to the desired concentration and mixed for infection.

^c^The pigs were infected with the bacterial mixture and monitored closely, and the surviving animals were euthanized 24 h after infection. Deep lung tissue was collected and homogenated, and bacteria were re-isolated. Single colonies were verified by PCR with primers specific for the genes apxIVA, fliY, and ydjN. Colonies positive in the amplification of apxIVA, fliY, and ydjN were recognized as WT, while colonies positive in apxIVA amplification but negative in fliY and ydjN were ΔfliYΔydjN. Colonies that showed other results should be confirmed and were not considered in the CI calculation.

^d^The CI value was calculated by dividing the ratio of ΔfliYΔydjN to WT in the output by the ratio of ΔfliYΔydjN to WT in the input. The CI value of < 0.2 was considered to be significantly attenuated.

## Discussion

As one of the key nutrients, sulfur possesses several singular features and is irreplaceable in biochemistry. Growing cells must find ways to continuously import sulfur at a high rate (Zhou and Imlay, 2020). The ability of pathogenic bacteria to obtain sulfur-containing nutrients in different host niches may therefore contribute to their virulence. For instance, the sulfate-binding protein CysP plays an important role in the growth and survival of *Moraxella catarrhalis* and is an excellent candidate vaccine antigen to prevent *M. catarrhalis* infection (Murphy et al., 2016). Elements involved in the acquisition of sulfur nutrients and their importance in the porcine respiratory tract pathogen, *A. pleuropneumoniae*, were investigated previously, and it was found that the sulfate-binding protein Sbp enables the utilization of sulfate and methionine. However, the *sbp* gene is not involved in the pathogenesis of *A. pleuropneumoniae* infection (Gao et al., 2020).

Cysteine is a sulfur-containing amino acid important for protein synthesis and maintaining enzymes and a metabolic precursor of many essential biomolecules and defense compounds, such as iron–sulfur clusters, vitamins, cofactors, and glutathione. Cysteine and cystine can be transformed into each other depending on the prevailing redox state (Kohlmeier, 2003). Several bacterial cysteine/cystine transporters have been identified, and their roles have been confirmed. In *E. coli*, the roles of the low-affinity symporter YdjN and the high-affinity ABC importer FliY in cysteine/cystine transport have been previously confirmed (Ohtsu et al., 2015; Sabrialabed et al., 2020), and structurally similar substances such as *S*-sulfocysteine are transported by YdjN (Yamazaki et al., 2016). Additionally, the involvement of these two transport systems in the uptake of toxic _L_-selenaproline and _L_-selenocystine makes these compounds an effective treatment of the urinary tract pathogen *E. coli* (Deutch et al., 2014). In this study, the potential cysteine/cystine transporters of *A. pleuropneumoniae* were named according to their *E. coli* counterparts FliY and YdjN, respectively. To better understand the role of FliY and YdjN in *A. pleuropneumoniae* sulfur utilization and virulence, we first characterized the growth of *fliY* and *ydjN* mutants. The growth levels of the *fliY* and *ydjN* single-gene deletion mutants were similar to the WT strain in both TSB and CDM, while the double-gene deletion mutant Δ*fliY*Δ*ydiN* was unable to grow in CDM with cysteine or cystine as the sole sulfur source. In addition, it was noted that the mutant Δ*fliY*Δ*ydiN* showed a longer lag phase in the TSB medium relative to that of the WT. A similar result was observed in our previous study, and the sulfate-binding protein Sbp mutant Δ*sbp* had a longer lag phase and lower growth levels when cultured in TSB compared to those of the WT (Gao et al., 2020). It is probable that these mutants take more time to adapt to other sulfur sources in TSB. These results indicate that both FliY and YdjN are involved in the cysteine/cystine-dependent growth of *A. pleuropneumoniae*.

The accumulation of oxidative active substances, such as superoxide and H_2_O_2_ generated from the electron transport chain and H_2_O_2_ produced by the host phagocytes, is harmful to bacteria cells, interfering with the normal redox state and causing damage to proteins, lipids, and nucleic acids of bacteria (Imlay, 2013). The sulfhydryl group of cysteine in the periplasm is one of the strategies for bacteria to diminish the toxicity of oxidative stress (Ohtsu et al., 2010). In addition, cysteine is an essential precursor for the biosynthesis of glutathione, a major antioxidant maintaining the homeostasis of the redox state in cells (Ku and Gan, 2021). However, cysteine is toxic to cells, since it may promote the Fenton reaction and generate harmful hydroxyl radicals (Imlay et al., 2015). Therefore, control of cysteine levels by coordinating its biosynthesis, utilization, oxidation, and transport is needed for bacterial defense against oxidative stress (Loddeke et al., 2017; Mironov et al., 2020). Mutation in *Lactobacillus fermentum* BspA, a basic surface-exposed protein homologous to FliY of *E. coli*, impaired bacterial cystine uptake significantly and showed an oxidation-sensitive phenotype, probably because the *bspA* mutant was unable to provide enough cystine for the production of the sulfhydryl compound to antagonize oxidative conditions (Turner et al., 1999). In addition, the cysteine transporter (YdeD) and cystine importers (YdjN and FliY-YecSC) in *E. coli* were reported to work cooperatively to keep reducing the equivalent in the periplasm, and the disruption of *ydeD* and *fliY* increases the sensitivity of membrane lipids to H_2_O_2_-induced oxidative damage (Ohtsu et al., 2015). Here, we found that mutation in the *A. pleuropneumoniae* cysteine/cystine transporters FliY and YdjN increased vulnerability to H_2_O_2_-induced oxidative stress. These results further emphasize the importance of cysteine/cystine transport for bacterial redox homeostasis.

Bacterial biofilms are defined as a community of surface-attached bacteria that are surrounded by hydrated polymeric matrixes of their own synthesis (Huigens et al., 2008). Bacterial biofilm formation is a complex multistep process involving several different factors (Alves-Barroco et al., 2020). Sulfur source utilization was indicated to play a role in biofilm formation in *Staphylococcus aureus* via the CymR-mediated regulation of the cysteine metabolism (Soutourina et al., 2009). In this study, we confirmed that the *A. pleuropneumoniae* wild-type stain (WF83) formed more biofilm in CDM than that in TSB. This is consistent with the previous view that the culture conditions were critical for the biofilm formation of *A. pleuropneumoniae* (Labrie et al., 2010). In addition, the expression of YdjN, but not FliY, on the shuttle vector pJFF224-XN (Δ*fliY*Δ*ydjN*-*ydjN*^+^ and WT-*ydjN*^+^) enhanced the *A. pleuropneumoniae* biofilm formation significantly in CDM. This finding sheds new light on the function of the cysteine/cystine transporter YdjN in biofilm formation. In addition, *N*-acetyl-_D_-glucosamine (GlcNAc) residues in β(1,6) linkage (PGA) catalyzed by the product of the *pgaC* gene has been shown to be a major biofilm adhesin of *A. pleuropneumoniae* (Izano et al., 2007; Liu et al., 2008). Surprisingly, the results indicated that the *A. pleuropneumoniae* strain WF83 (*pgaC* mutant, Liu et al., 2008) and its derivatives were able to form biofilms in CDM. It is possible that YdjN is involved in controlling biofilm formation via a PGA-independent mechanism in *A. pleuropneumoniae*.

The role of sulfur source uptake and metabolism in bacterial virulence has been documented previously. In *S. aureus*, TcyABC and TcyP involved in the transport of cystine, cysteine, and *N*-acetyl cysteine are necessary for *in vivo* colonization and affect virulence (Lensmire et al., 2020). FliY and YdjN are essential for *A. pleuropneumoniae* growth using cysteine or cystine as a sole sulfur source. In addition, these transporters promote *A. pleuropneumoniae* cells to oxidative stress tolerance. Therefore, we hypothesized that FliY and YdjN should also be related to virulence. To test this hypothesis, the virulence of the *A. pleuropneumoniae* WT and Δ*fliY*Δ*ydjN* was evaluated in a mouse infection model and its natural host, the pig. However, there was no significant decrease in the bacterial loads in the lung tissues of the mice, and the competitive index of the mutant relative to the WT in the lung tissue of the pigs was above the threshold value of attenuation. These observations clearly indicate that the transporters FliY and YdjN are not correlated with the *in vivo* colonization and bacterial virulence of *A. pleuropneumoniae*. A similar result was observed in our previous study, in which the *A. pleuropneumoniae* mutant Δ*sbp* was shown to be defective in using sulfate and methionine but still virulent (Gao et al., 2020). Probably, other sulfur nutrients instead of cystine/cysteine support the growth of Δ*fliY*Δ*ydjN* in the host. Furthermore, oxidative resistance conferred by other proteins, such as LonA (Xie et al., 2016), TolC2 (Li et al., 2017), TolC1 (Li et al., 2019), FtpA (Tang et al., 2022), and HtrA (Zhang et al., 2022), may protect *A. pleuropneumoniae* cysteine/cystine uptake mutant from oxidative toxicity. These results further demonstrate the complex mechanisms involved in the pathogenesis of *A. pleuropneumoniae* infection.

In conclusion, the present study shows that FliY and YdjN are important for the uptake of cysteine/cystine as sulfur sources. This study demonstrates that FliY and YdjN are related to oxidative stress tolerance, and YdjN might be a potential regulator of biofilm formation in *A. pleuropneumoniae*. The FliY- and YdjN-dependent sulfur nutrient satisfaction and oxidative stress response are not correlated with the pathogenicity of *A. pleuropneumoniae*.

## Data availability statement

The original contributions presented in the study are included in the article/Supplementary material, further inquiries can be directed to the corresponding authors.

## Ethics statement

The animal study was reviewed and approved by the Animal Ethics Committee at the Central China Normal University. Written informed consent was obtained from the owners for the participation of their animals in this study.

## Author contributions

JL and CQ designed the research, provided the experiment conditions, and helped with the data analysis. FZ and JL wrote the manuscript. FZ, HX, YC, JX, and MZ performed the experiments. FZ, HX, YC, and ZL contributed to the animal experiments. All authors reviewed and approved the manuscript.

## Supplementary material

Supplementary Figure S1 Verification of double-gene deletion mutant Δ*fliY*Δ*ydjN*. **(A)** PCR amplification with primers *fliY*-F1 and *fliY*-R2. Lane M, DNA ladder 2000; lanes 1–3, Δ*fliY*Δ*ydjN*; lane 4, vector pEMOC2-Δ*fliY*; lane 5, WT; lane 6, negative control. **(B)** PCR amplification with primers *ydjN*-F5 and *ydjN*-R5. Lane M, DNA ladder 2000; lanes 1–4, Δ*fliY*Δ*ydjN*; lane 5, vector pEMOC2-Δ*ydjN*; lane 6, WT; lane 7, negative control.

Supplementary Figure S2 Identification of complementation strains. **(A)** Δ*fliY*Δ*ydiN*-*fliY*^+^ verified with primers *fliY*-F3 and *fliY*-R3. Lane M, DNA ladder 2000; lane 1, negative control; lane 2, WT; lanes 3–5, Δ*fliY*Δ*ydiN*-*fliY*^+^. **(B)** Δ*fliY*Δ*ydiN*-*ydjN*^+^ verified with primers *ydjN*-F3 and *ydjN*-R3. Lane M, DNA ladder 2000; lane 1, negative control; lane 2, WT; lane 3, Δ*fliY*Δ*ydiN*-*ydjN*^+^. **(C)** RT-PCR assay with primers *fliY*-F4 and *fliY*-R4. Lane M, DNA ladder 2000; lanes 1–3, Δ*fliY*Δ*ydiN*-*fliY*^+^; lane 4, WT; lane 5, Δ*fliY*Δ*ydiN*; lane 6, negative control. **(D)** RT-PCR assay with primers *ydjN*-F4 and *ydjN*-R4. Lane M, DNA ladder 2000; lanes 1 and 2, negative control; lane 3, WT; lane 4, Δ*fliY*Δ*ydiN*; lanes 5–8, Δ*fliY*Δ*ydiN*-*ydjN*^+^.

## References

[B1] Alves-Barroco C. Paquete-Ferreira J. Santos-Silva T. Fernandes A. R. (2020). Singularities of pyogenic Streptococcal biofilms - from formation to health implication. Front. Microbiol. 11, 584947. 10.3389/fmicb.2020.584947 33424785 PMC7785724

[B2] Baltes N. Hennig-Pauka I. Gerlach G. F. (2002). Both transferrin binding proteins are virulence factors in *Actinobacillus pleuropneumoniae* serotype 7 infection. FEMS Microbiol. Lett. 209, 283–287. 10.1111/j.1574-6968.2002.tb11145.x 12007819

[B3] Beddek A. J. Sheehan B. J. Bossé J. T. Rycroft A. N. Kroll J. S. Langford P. R.. (2004). Two TonB systems in *Actinobacillus pleuropneumoniae*: their roles in iron acquisition and virulence. Infect. Immun. 72, 701–708. 10.1128/IAI.72.2.701-708.2004 14742511 PMC321588

[B4] Beinert H. (2000). A tribute to sulfur. Eur. J. Biochem. 267, 5657–5664. 10.1046/j.1432-1327.2000.01637.x 10971575

[B5] Chiers K. De Waele T. Pasmans F. Ducatelle R. Haesebrouck F. (2010). Virulence factors of *Actinobacillus pleuropneumoniae* involved in colonization, persistence and induction of lesions in its porcine host. Vet. Res. 41, 65. 10.1051/vetres/2010037 20546697 PMC2899255

[B6] Deutch C. E. Spahija I. Wagner C. E. (2014). Susceptibility of *Escherichia coli* to the toxic _L_-proline analogue _L_-selenaproline is dependent on two _L_-cystine transport systems. J. Appl. Microbiol. 117, 1487–1499. 10.1111/jam.12623 25139244

[B7] Frey J. (1992). Construction of a broad host range shuttle vector for gene cloning and expression in *Actinobacillus pleuropneumoniae* and other Pasteurellaceae. Res. Microbiol. 143, 263–269. 10.1016/0923-2508(92)90018-J 1448612

[B8] Gao L. Zhang L. Xu H. Zhao F. Ke W. Chen J.. (2020). The *Actinobacillus pleuropneumoniae* sulfate-binding protein is required for the acquisition of sulfate and methionine, but is not essential for virulence. Vet. Microbiol. 245, 108704. 10.1016/j.vetmic.2020.108704 32456813

[B9] Herriott R. M. Meyer E. Y. Vogt M. Modan M. (1970). Defined medium for growth of *Haemophilus influenzae*. J. Bacteriol. 101, 513–516. 10.1128/jb.101.2.513-516.1970 5308770 PMC284935

[B10] Hicks J. L. Mullholland C. V. (2018). Cysteine biosynthesis in *Neisseria species*. Microbiology 164, 1471–1480. 10.1099/mic.0.000728 30307392

[B11] Huigens R. W. 3rd. Ma L. Gambino C. Moeller P.D.R. Basso A. Cavanagh J. Wozniak D.J. Melander C. (2008). Control of bacterial biofilms with marine alkaloid derivatives. Mol. Biosyst. 4, 614–621. 10.1039/b719989a 18493660

[B12] Imlay J. A. (2013). The molecular mechanisms and physiological consequences of oxidative stress: lessons from a model bacterium. Nat. Rev. Microbiol. 11, 443–454. 10.1038/nrmicro3032 23712352 PMC4018742

[B13] Imlay K. R. C. Korshunov S. Imlay J. A. (2015). Physiological roles and adverse effects of the two cystine importers of *Escherichia coli*. J. Bacteriol. 197, 3629–3644. 10.1128/JB.00277-15 26350134 PMC4626903

[B14] Izano E. A. Sadovskaya I. Vinogradov E. Mulks M. H. Velliyagounder K. Ragunath C.. (2007). Poly-N-acetylglucosamine mediates biofilm formation and antibiotic resistance in *Actinobacillus pleuropneumoniae*. Microb. Pathog. 43, 1–9. 10.1016/j.micpath.2007.02.004 17412552 PMC1950449

[B15] Kohlmeier M. (2003). “Cysteine,” in Nutrient Metabolism, ed. M. Kohlmeier (London: Academic Press), 348–356. 10.1016/B978-012417762-8.50055-7

[B16] Ku J. W. K. Gan Y. H. (2021). New roles for glutathione: modulators of bacterial virulence and pathogenesis. Redox Biol. 44, 102012. 10.1016/j.redox.2021.102012 34090244 PMC8182430

[B17] Labrie J. Pelletier-Jacques G. Deslandes V. Ramjeet M. Auger E. Nash J. H.. (2010). Effects of growth conditions on biofilm formation by *Actinobacillus pleuropneumoniae*. Vet. Res. 41, 3. 10.1051/vetres/2009051 19737507 PMC2762130

[B18] Lensmire J. M. Dodson J. P. Hsueh B. Y. Wischer M. R. Delekta P. C. Shook J. C.. (2020). The *Staphylococcus aureus* cystine transporters TcyABC and TcyP facilitate nutrient sulfur acquisition during infection. Infect. Immun. 88, e00690–19. 10.1128/IAI.00690-19 31843961 PMC7035926

[B19] Li Y. Cao S. Zhang L. Yuan J. Yang Y. Zhu Z.. (2017). TolC2 is required for the resistance, colonization and virulence of *Actinobacillus pleuropneumoniae*. J. Med. Microbiol. 66, 1170–1176. 10.1099/jmm.0.000544 28758624

[B20] Li Y. Cao S. Zhang L. Yuan J. Zhao Q. Wen Y.. (2019). A requirement of TolC1 for effective survival, colonization and pathogenicity of *Actinobacillus pleuropneumoniae*. Microb. Pathog. 134, 103596. 10.1016/j.micpath.2019.103596 31212036

[B21] Liu J. Cao Y. Gao L. Zhang L. Gong S. Yang J.. (2018). Outer membrane lipoprotein Lip40 modulates adherence, colonization, and virulence of *Actinobacillus pleuropneumoniae*. Front. Microbiol. 9, 1472. 10.3389/fmicb.2018.01472 30018613 PMC6038445

[B22] Liu J. Stone V. N. Ge X. Tang M. Elrami F. Xu P.. (2017). TetR family regulator *brpT* modulates biofilm formation in *Streptococcus sanguinis*. PLoS ONE 21, e0169301. 10.1371/journal.pone.0169301 28046010 PMC5207742

[B23] Liu J. Tan C. Li J. Chen H. Xu P. He Q.. (2008). Characterization of ISApl*1*, an insertion element identified from *Actinobacillus pleuropneumoniae* field isolate in China. Vet. Microbiol. 132, 348–354. 10.1016/j.vetmic.2008.05.031 18632228

[B24] Loddeke M. Schneider B. Oguri T. Mehta I. Xuan Z. Reitzer L.. (2017). Anaerobic cysteine degradation and potential metabolic coordination in *Salmonella enterica* and *Escherichia coli*. J. Bacteriol. 199, e00117–17. 10.1128/JB.00117-17 28607157 PMC5527379

[B25] Méndez J. Reimundo P. Pérez-Pascual D. Navais R. Gómez E. Guijarro J. A.. (2011). A novel *cdsAB* operon is involved in the uptake of _L_-cysteine and participates in the pathogenesis of *Yersinia ruckeri*. J. Bacteriol. 193, 944–951. 10.1128/JB.01058-10 21169490 PMC3028680

[B26] Mironov A. Seregina T. Shatalin K. Nagornykh M. Shakulov R. Nudler E.. (2020). CydDC functions as a cytoplasmic cystine reductase to sensitize *Escherichia coli* to oxidative stress and aminoglycosides. Proc. Natl. Acad. Sci. USA. 117, 23565–23570. 10.1073/pnas.2007817117 32900959 PMC7519257

[B27] Murphy T. F. Kirkham C. Johnson A. Brauer A. L. Koszelak-Rosenblum M. Malkowski M. G.. (2016). Sulfate-binding protein, CysP, is a candidate vaccine antigen of *Moraxella catarrhalis*. Vaccine 34, 3855–3861. 10.1016/j.vaccine.2016.05.045 27265455

[B28] Núñez G. Sakamoto K. Soares M. P. (2018). Innate nutritional immunity. J. Immunol. 201, 11–18. 10.4049/jimmunol.1800325 29914937 PMC6028930

[B29] Ohtsu I. Kawano Y. Suzuki M. Morigasaki S. Saiki K. Yamazaki S.. (2015). Uptake of _L_-cystine via an ABC transporter contributes defense of oxidative stress in the _L_-cystine export-dependent manner in *Escherichia coli*. PLoS ONE 10, e0120619. 10.1371/journal.pone.0120619 25837721 PMC4383340

[B30] Ohtsu I. Wiriyathanawudhiwong N. Morigasaki S. Nakatani T. Kadokura H. Takagi H.. (2010). The _L_-cystine/_L_-cystine shuttle system provides reducing equivalents to the periplasm in *Escherichia coli*. J. Biol. Chem. 285, 17479–17487. 10.1074/jbc.M109.081356 20351115 PMC2878512

[B31] Oswald W. Tonpitak W. Ohrt G. Gerlach G. (1999). A single-step transconjugation system for the introduction of unmarked deletions into *Actinobacillus pleuropneumoniae* serotype 7 using a sucrose sensitivity marker. FEMS Microbiol. Lett. 179, 153–160. 10.1111/j.1574-6968.1999.tb08721.x 10481100

[B32] Paritala H. Carroll K. S. (2013). New targets and inhibitors of mycobacterial sulfur metabolism. Infect. Disord. Drug Targets 13, 85–115. 10.2174/18715265113139990022 23808874 PMC4332622

[B33] Sabrialabed S. Yang J. G. Yariv E. Ben-Tal N. Lewinson O. (2020). Substrate recognition and ATPase activity of the *E. coli* cysteine/cystine ABC transporter YecSC-FliY. J. Biol. Chem. 295, 5245–5256. 10.1074/jbc.RA119.012063 32144203 PMC7170509

[B34] Sassu E. L. Bossé J. T. Tobias T. J. Gottschalk M. Langford P. R. Hennig-Pauka I.. (2018). Update on *Actinobacillus pleuropneumoniae*-knowledge, gaps and challenges. Transbound. Emerg. Dis. 65, 72–90. 10.1111/tbed.12739 29083117

[B35] Schaller A. Djordjevic S. P. Eamens G. J. Forbes W. A. Kuhn R. Kuhnert P.. (2001). Identification and detection of *Actinobacillus pleuropneumoniae* by PCR based on the gene *apxIVA*. Vet. Microbiol. 79, 47–62. 10.1016/S0378-1135(00)00345-X 11230928

[B36] Soutourina O. Poupel O. Coppe J. Y. Danchin A. Msadek T. Martin-Verstraete I.. (2009). CymR, the master regulator of cysteine metabolism in *Staphylococcus aureus*, controls host sulphur source utilization and plays a role in biofilm formation. Mol. Microbiol. 73, 194–211. 10.1111/j.1365-2958.2009.06760.x 19508281

[B37] Tang H. Zhang Q. Han W. Wang Z. Pang S. Zhu H.. (2022). Identification of FtpA, a Dps-like protein involved in anti-oxidative stress and virulence in *Actinobacillus pleuropneumoniae*. J. Bacteriol. 204, e0032621. 10.1128/jb.00326-21 34807725 PMC8846326

[B38] Turner M. S. Woodberry T. Hafner L. M. Giffard P. M. (1999). The *bspA* locus of *Lactobacillus fermentum* BR11 encodes an _L_-cystine uptake system. J. Bacteriol. 181, 2192–2198. 10.1128/JB.181.7.2192-2198.1999 10094698 PMC93633

[B39] Xie F. Li G. Zhang Y. Zhou L. Liu S. Liu S.. (2016). The Lon protease homologue LonA, not LonC, contributes to the stress tolerance and biofilm formation of *Actinobacillus pleuropneumoniae*. Microb. Pathog. 93, 38–43. 10.1016/j.micpath.2016.01.009 26796296

[B40] Yamazaki S. Takei K. Nonaka G. (2016). *ydjN* encodes an *S*-sulfocysteine transporter required by *Escherichia coli* for growth on *S*-sulfocysteine as a sulfur source. FEMS Microbiol. Lett. 363, fnw185. 10.1093/femsle/fnw185 27481704

[B41] Zhang L. Zhao F. Xu H. Chen Y. Qi C. Liu J.. (2022). HtrA of *Actinobacillus pleuropneumoniae* is a virulence factor that confers resistance to heat shock and oxidative stress. Gene 841, 146771. 10.1016/j.gene.2022.146771 35905850

[B42] Zhou Y. Imlay J. A. (2020). *Escherichia coli* K-12 lacks a high-affinity assimilatory cysteine importer. MBio 11, e01073–20. 10.1128/mBio.01073-20 32518189 PMC7373191

