# Supplementary figures and images for: Actinobacillus pleuropneumoniae FliY and YdjN are involved in cysteine/cystine utilization, oxidative resistance, and biofilm formation but are not determinants of virulence
Source: Front Microbiol. 2023 May 12;14:1169774. doi: 10.3389/fmicb.2023.1169774 (PMC10213525; doi:10.3389/fmicb.2023.1169774)

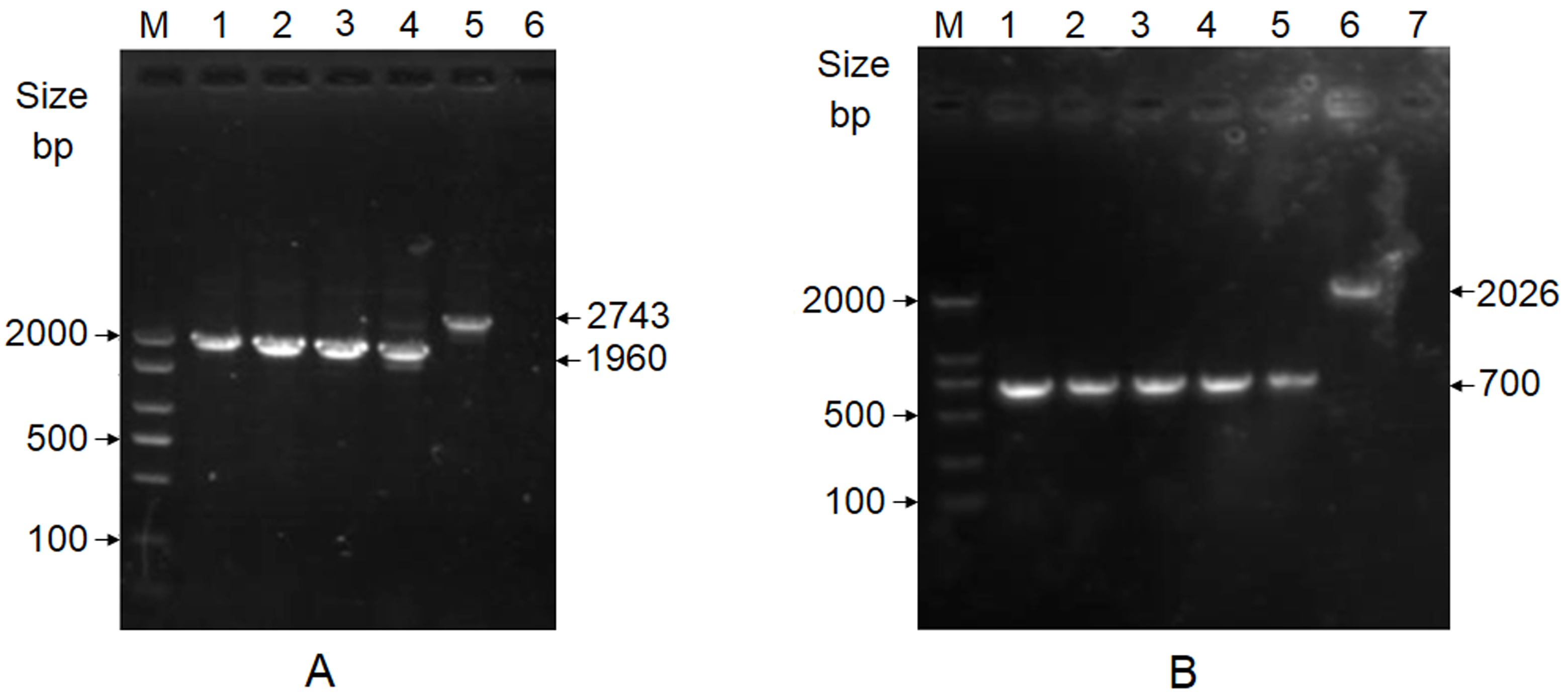

Supplement: Supplementary Figure S1 — Verification of double-gene deletion mutant ΔfliYΔydjN. (A) PCR amplification with primers fliY-F1 and fliY-R2. Lane M, DNA ladder 2000; lanes 1–3, ΔfliYΔydjN; lane 4, vector pEMOC2-ΔfliY; lane 5, WT; lane 6, negative control. (B) PCR amplification with primers ydjN-F5 and ydjN-R5. Lane M, DNA ladder 2000; lanes 1–4, ΔfliYΔydjN; lane 5, vector pEMOC2-ΔydjN; lane 6, WT; lane 7, negative control. [file Image_1.TIF]

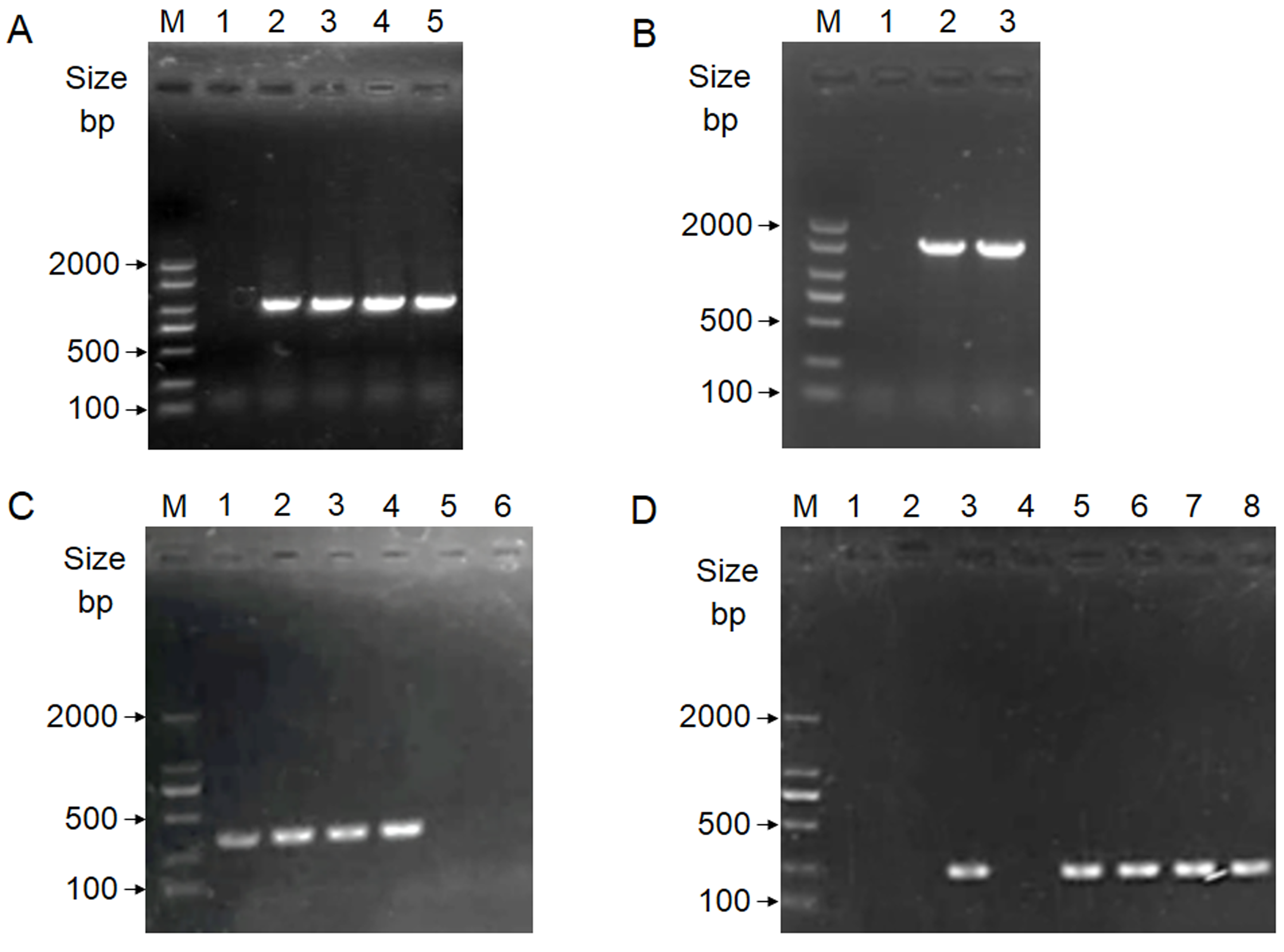

Supplement: Supplementary Figure S2 — Identification of complementation strains. (A) ΔfliYΔydiN-fliY+ verified with primers fliY-F3 and fliY-R3. Lane M, DNA ladder 2000; lane 1, negative control; lane 2, WT; lanes 3–5, ΔfliYΔydiN-fliY+. (B) ΔfliYΔydiN-ydjN+ verified with primers ydjN-F3 and ydjN-R3. Lane M, DNA ladder 2000; lane 1, negative control; lane 2, WT; lane 3, ΔfliYΔydiN-ydjN+. (C) RT-PCR assay with primers fliY-F4 and fliY-R4. Lane M, DNA ladder 2000; lanes 1–3, ΔfliYΔydiN-fliY+; lane 4, WT; lane 5, ΔfliYΔydiN; lane 6, negative control. (D) RT-PCR assay with primers ydjN-F4 and ydjN-R4. Lane M, DNA ladder 2000; lanes 1 and 2, negative control; lane 3, WT; lane 4, ΔfliYΔydiN; lanes 5–8, ΔfliYΔydiN-ydjN+. [file Image_2.TIF]
